# Lymphohematopoietic cancer mortality among Korean semiconductor manufacturing workers

**DOI:** 10.1186/s12889-023-16325-z

**Published:** 2023-08-02

**Authors:** Dong-Wook Lee, Sooyoung Cho, Aesun Shin

**Affiliations:** 1 Department of Occupational and Environmental Medicine, Inha University Hospital, Inha University, Incheon, Republic of Korea; 2 Department of Preventive Medicine, Seoul National University College of Medicine, Seoul, Republic of Korea; 3 Genomic Medicine Institute, Seoul National University Medical Research Center, Seoul, Republic of Korea; 4 Cancer Research Institute, Seoul National University, Seoul, Republic of Korea

**Keywords:** Lymphohematopoietic cancer, Semiconductor industry, Occupational disease, Leukemia

## Abstract

**Background:**

We aimed to examine the lymphohematopoietic cancer mortality in a cohort of workers at a semiconductor manufacturing company in South Korea according to their jobs.

**Methods:**

A retrospective cohort was constructed using the personnel records of semiconductor manufacturing workers who were employed in a semiconductor company in South Korea in 1998–2012. Data on their vital status and causes of death were obtained from the National Statistical Office of South Korea. The standardized mortality ratios (SMRs) of lymphohematopoietic cancer were calculated.

**Results:**

A total of 288 deaths were reported, of which 22 were caused by lymphohematopoietic cancer, among 65,782 workers in 878,325 person-years. The SMRs for lymphohematopoietic cancer were 0.78 (95% confidence interval [CI] = 0.39–1.40; the number of observed cases [Obs] = 11) among male workers and 1.71 (95% CI = 0.85–3.06; Obs = 11) among female workers. Among female operators, excess deaths due to lymphohematopoietic cancer (SMR = 2.59, 95% CI = 1.24–4.76) and leukemia (SMR = 2.92, 95% CI = 1.26–5.76) were observed. However, they were not observed among office workers, facility managers, utility managers, or process managers.

**Conclusion:**

Female operators involved in the semiconductor wafer fabrication process had higher risk of mortality from lymphohematopoietic cancer.

**Supplementary Information:**

The online version contains supplementary material available at 10.1186/s12889-023-16325-z.

## Background

Lymphohematopoietic cancers such as leukemia, lymphomas, and myelomas are diverse types of neoplasms that arise from various stem cells at different hierarchical levels of hematopoietic and lymphoid cell development [1]. Leukemia is a common subgroup of lymphohematopoietic cancers that causes an increase in the number of leukocytes in the blood and bone marrow. Globally, leukemia causes 0.4 million incident cases and 0.3 million deaths [2]. In South Korea, the number of leukemia cases was 3,605 in 2020, accounting for 1.5% of the total cancer cases [3]. The occupational risk factors of leukemia have been investigated. The International Agency for Research on Cancer has suggested occupational carcinogens such as benzene, ethylene oxide, 1,3-butadiene, ionizing radiation, and boot and shoe manufacturing and repair as risk factors for leukemia and non-Hodgkin’s lymphoma (NHL) [4]. The population-attributable risk of occupational risk factors for leukemia was 2% [5].

In South Korea, a 22-year-old female worker who had worked in the semiconductor manufacturing plants died due to acute myeloid leukemia in 2007. This story was covered by the media and gained public attention, through the efforts of a workers’ support group and her family [6]. The Occupational Safety and Health Research Institute of South Korea investigated all workers from all five semiconductor companies for a cancer cluster, and concluded that the incidence of or mortality from leukemia did not increase [7]. A related case series of seven workers with lymphohematopoietic cancer [four acute myeloid leukemia, one acute lymphoblastic leukemia, one NHL, and one aplastic anemia] among semiconductor companies also reported that the level of exposure to occupational hazards, including ionizing radiation, airborne benzene, ethylene oxide, and formaldehyde, was extremely low and were not considered as causes of disease [8].

Semiconductor manufacturing can be broadly classified into fabrication processes, manufacturing semiconductor integrated circuits on wafers, assembly processes, and packaging of processed wafers into individual chip units. The fabrication process consists of photoresist application, photoexposure, etching, deposition, ion implantation, and chemical or mechanical polarization. In addition to the degree of protection, all fabrication processes involve the use of various hazardous chemicals or harmful physical factors [9, 10]. Therefore, cancer risk in the semiconductor industry has been of constant concern in several countries owing to the thousands of different chemicals and physical hazards used in the semiconductor industry, including organic solvents that are utilized in the wafer fabrication process [11].

The semiconductor industry in South Korea has significantly expanded over the past 30 years. During this period, the semiconductor manufacturing process changed rapidly and considerably, followed by changes in the use of chemicals and facilities, including protection systems. In particular, the wafer fabrication process is a potential hazard to the workers’ health owing to the use of harmful chemicals and ionizing radiation; however, the working environment was rapidly altered during this period. This makes it difficult for researchers to determine whether the wafer-fabrication process is hazardous.

Over several decades, the potential hazards of the semiconductor industry have increased, but epidemiological studies have reported no significant excess cancer incidence or mortality among semiconductor workers in the United Kingdom (UK), the United States (US), Taiwan, and South Korea [12–16]. A systematic review and meta-analysis also reported no significant association between semiconductor work and leukemia [17].

Therefore, a well-investigated study is warranted to determine the relationship between excess lymphohematopoietic cancer mortality and the semiconductor industry, especially the wafer fabrication process, in South Korea. We investigated the cancer mortality among those who worked at a semiconductor company by department, job classification, and working period and evaluated the associations between cancer and work in the wafer fabrication process.

## Methods

### Cohort definition

**Fig. 1:**
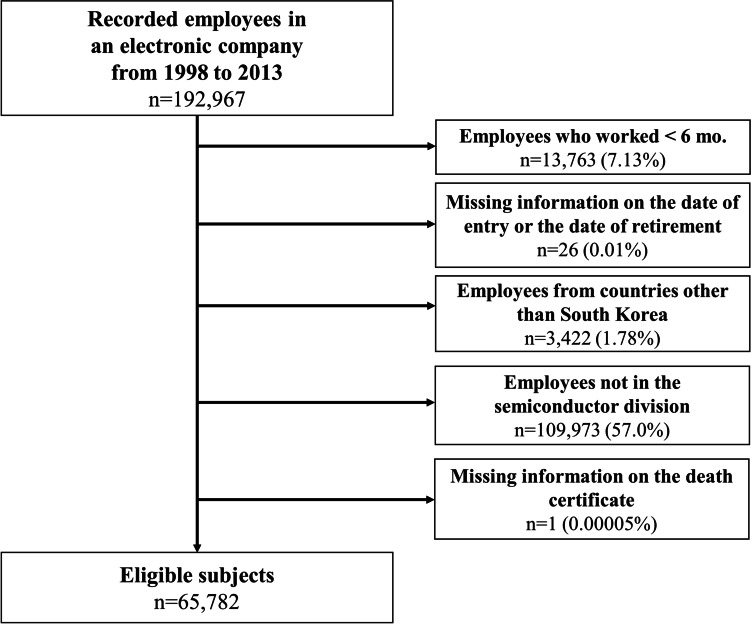
Schematic diagram depicting the participant selection process

The study population comprised employees of an electronic company in South Korea. The cohort included employees who had worked in the part of the company related to semiconductor manufacturing between January 1, 1998, and December 31, 2012. The follow-up of employees was started in 1998 as the company’s employee records only included information documented from the period of 1998. Human resource records of current and former employees from the company were obtained, including personal identification numbers, birth date, sex, hiring date, education status, department and job classification on the last day of every year, retirement date, and vital status. We excluded employees who had worked at the company for less than 6 months (*n* = 13,763). The minimum length of employment criterion was chosen according to that indicated in the previous study reporting the cancer mortality of US workers employed in the semiconductor industry [15]. Participants with missing information on the date of entry or retirement were excluded (*n* = 26). Employees from countries other than South Korea were also excluded as it was impossible to obtain the data on their vital status (*N* = 3,422). Workers not in the semiconductor manufacturing division were excluded (*n* = 109,973). One employee who died earlier than the date of entry was excluded. Overall, only 65,782 workers were enrolled in the final study. The participant selection process is shown in Fig. 1. This study was approved by the Institutional Review Board of Seoul National University Hospital (C-1708–159-880).

### Job classification

The departments and jobs of the workers in the semiconductor division were provided by the company’s Department of Human Resources. The classification of departments and jobs in a company’s semiconductor division has changed continuously because of the rapidly changing semiconductor industry. Because classifications, departments, and jobs differed in name and role even after 1 year, they were reviewed and reclassified using the annual human resource record data. Finally, the employees were classified according to the seven job categories (Additional file 1): 1) workers in the non-semiconductor division (working in divisions other than the semiconductor division, such as the hard disk drive division, optical and mechanical solution division, and storage division), 2) office workers in the semiconductor division, 3) operator (operating facilities to produce semiconductor products or testing for acceptance and inspecting defective products), 4) facility engineer (being assigned to specific manufacturing facilities for inspection and maintenance), 5) utility manager (establishing and providing infrastructure for semiconductor production at the “whole-plant level”), 6) process engineer (maintaining and improving the manufacturing process to improve the yield and quality of products), and 7) not classifiable (cannot be included anywhere among the other six categories). If the job classification changed during the follow-up period, the most prolonged worked job classification was used as the representative category.

### Determination of vital status and cause of death

The vital status from January 1, 1998 to December 31, 2015 was determined using the National Statistical Office (NSO) database of South Korea. The NSO has provided an annual report on the cause-of-death statistics of South Koreans since 1982 and presents a record linkage service between national death certificate files and personal identification. The NSO data covered 99.7% of the total deaths [18]. Human resource data were sent to link the personal identification number of each employee to the NSO cause-of-death data, and data on the vital status, cause of death, and date of death were obtained. Unmatched employees were considered alive at the end of the follow-up period (December 31, 2015). The causes of death were coded according to the International Classification of Diseases, 10th revision (ICD-10). All-cause death was defined as death status in the NSO data. The cancer mortality cases were classified according to the ICD-10 code indicated in the cause-of-death data: malignant neoplasm (C00–C97); lymphoid, hematopoietic, and related tissues (C81–C96); leukemia (C91, C95); and non-Hodgkin’s Lymphoma (C82–C85).

### Statistical analysis

We calculated the standardized mortality ratios (SMRs) to compare the mortality rate of the study cohort with that of the general population of South Korea. Indirect standardization is a statistical method frequently implemented in epidemiology for comparing mortality rates across different populations, while adjusting for age and sex distribution. The standardized mortality ratio (SMR) represents the ratio of observed deaths to expected deaths. First, the follow-up duration of the participants was summed according to the 5-year age groups and sex. The number of expected cases in the study population was calculated using the summed up follow-up duration, the reference mortality rate associated with the specific causes by age group, and sex-specific data provided by the NSO. The reference mortality rates by cause of death in the South Korean population for the median year (2006) of the follow-up period (1998–2015) were used. The reference mortality rates for various categories including all-cause mortality, malignant neoplasms (C00–C97), leukemia (C91-C95), and lymphoid, hematopoietic, and related tissue neoplasms (C81–C96) in 2006 were obtained by the National Statistics Office of Korea [19]. These rates were stratified by 5-year age groups and sex. To calculate the expected number of cases, the reference mortality rates were applied to the duration of follow-up for each participant. Finally, SMRs were calculated by dividing the number of observed cases by the number of expected cases. The SMRs for all-cause mortality, malignant neoplasm (C00–C97), leukemia (C91-C95), and lymphoid, hematopoietic, and related tissue neoplasms (C81–C96) were calculated. The *p*-values and 95% confidence intervals (95% CIs) were estimated using appropriate methods based on the Poisson distribution [20]. The results were presented according to sex and job category. Additionally, the SMRs for lymphhematopoietic cancers in female operators were calculated according to their first working year before 2005 and after 2005, the periods when important changes in the manufacturing environment were reported [21]. Finally, we summarized the characteristics of the observed deaths due to lymphoid, hematopoietic, and related tissue neoplasms (C81–C96), such as sex, job classification, date of entry, working duration, date of death, age at death, and cause of death. Statistical analyses were performed using SAS STDRATE procedures (version 9.3; SAS Institute Inc., Cary, NC, USA).

## Results

Table 1 shows the characteristics of Korean workers in semiconductor factories who worked for more than 6 months between 1998 and 2012. Among the 65,782 workers, 33,368 (50.7%) were men and 32,414 (49.3%) were women. Approximately 44.8% of male workers were born between 1970 and 1979, while 68.5% of female workers were born between 1980 and 1989. According to job classification, male workers were classified as office workers in the semiconductor factories (39.2%), facility engineers (23.7%), process engineers (15.8%), workers in the non-semiconductor division (13.5%), utility managers (2.7%), and operators (2.2%). Approximately 61.5% of female workers were classified as operators, 13.6% as workers in the non-semiconductor division, and 9.5% as office workers in the semiconductor factory.

**Table 1:** Characteristics of South Korean workers in the semiconductor factories, 1998–2014

	Total	Male workers	Female workers
	n (%)	n (%)	n (%)
Total	65,782 (100.0)	33,368 (100.0)	32,414 (100.0)
Birth year			
1930–1939	1 (0.002)	1 (0.003)	0 (0)
1940–1949	37 (0.1)	37 (0.1)	0 (0)
1950–1959	689 (1.1)	687 (2.1)	2 (0.006)
1960–1969	5,814 (8.8)	5,725 (17.2)	89 (0.3)
1970–1979	22,367 (34.0)	14,940 (44.8)	7,427 (22.9)
1980–1989	34,104 (51.8)	11,896 (35.7)	22,208 (68.5)
1990–1999	2,770 (4.2)	82 (0.3)	2,688 (8.3)
Job classification			
Workers in the non-semiconductor division	8,904 (13.5)	4,512 (13.5)	4,392 (13.6)
Office workers in the semiconductor division	16,171 (24.6)	13,079 (39.2)	3,092 (9.5)
Operator	20,657 (31.4)	736 (2.2)	19,921 (61.5)
Facility engineer	7,996 (12.2)	7,891 (23.7)	105 (0.3)
Utility manager	936 (1.4)	911 (2.7)	25 (0.1)
Process engineer	6,151 (9.4)	5,273 (15.8)	878 (2.7)
Not classifiable	4,967 (7.6)	966 (2.9)	4,001 (12.3)

A total of 288 deaths were reported among 65,782 workers aged > 878,325 person-years (Table 2). Of the total study population, 33,368 (50.7%) and 32,414 (49.3%) were male and female workers, respectively. The mean [± standard deviation (SD)] of the follow-up period was 13.3 (± 4.8) years (range: 0.5–19.0 years). The mean age (± SD) of the participants at study enrollment was 23.7 (± 6.0) years; the mean ages of the male and female workers were 27.5 (± 5.2) and 19.7 (± 2.2), respectively. The SMRs for all-cause mortality were 0.24 (95% CI = 0.21–0.28) in male workers and 0.71 (95% CI = 0.58–0.84) in female workers. The rate of excess death due to malignant neoplasms was significantly lower in male workers (SMR = 0.37, 95% CI = 0.28–0.48), but not in female workers (SMR = 1.20, 95% CI = 0.88–1.59). The SMRs of the malignant neoplasms in the lymphoid, hematopoietic, and related tissues were 0.78 (95% CI = 0.39–1.40) in male workers, with 11 observed deaths, and 1.71 (95% CI = 0.85–3.06) in female workers, with 11 observed deaths.

**Table 2:** Observed and expected deaths, SMRs, and 95% CIs for all causes and malignant neoplasms

	Male workers				Female workers			
Disease group (ICD-10)	Obs	Exp	SMR	95% CI	Obs	Exp	SMR	95% CI
All-cause death	169	697.9	0.24	(0.21 – 0.28)*	119	168.8	0.71	(0.58 – 0.84)*
(C00–C97) Malignant neoplasm	57	153.7	0.37	(0.28 – 0.48)*	47	39.3	1.20	(0.88 – 1.59)
(C81–C96) Lymphoid, hematopoietic, and related tissues	11	14.1	0.78	(0.39 – 1.40)	11	6.4	1.71	(0.85 – 3.06)
(C91–C95) Leukemia	10	8.3	1.20	(0.58 – 2.21)	8	4.5	1.77	(0.76 – 3.49)
(C82–C85) Non-Hodgkin’s lymphoma	1	4.5	0.22	(0.01 – 1.24)	3	1.4	2.10	(0.43 – 6.15)

*ICD-10 *the International Classification of Diseases 10th revision,* Obs* observed number of deaths, *Exp* expected number of deaths, *SMR* standardized mortality ratio, *CI* confidence interval

^*^p < 0.05

Table 3 shows the SMRs and 95% CIs for malignant neoplasms in the lymphoid, hematopoietic, and related tissues according to job classification in male and female workers. Most of the male workers were office workers in the semiconductor factors (*N* = 13,709, person-year = 172,998), and none of them experienced excess mortality due to malignant neoplasms in the lymphoid, hematopoietic, and related tissues (SMR = 0.85, 95% CI = 0.27–1.99). Most female workers were operators (*N* = 19,921; person-years = 267,371). Among female operators, significant excess mortality due to malignant neoplasms in the lymphoid, hematopoietic, and related tissues was observed (SMR = 2.59, 95% CI = 1.24–4.76); ten patients died, of whom eight died due to leukemia (SMR = 2.92, 95% CI = 1.26–5.76) and two died due to NHL (SMR = 2.42, 95% CI = 0.29–8.75).

**Table 3:** Observed and expected deaths, SMRs, and 95% Cls for malignant neoplasms in the lymphoid, hematopoietic, and related tissues (C81-C96) by job classification

Job classification	**Male workers**						**Female workers**					
	***N***	**Person-year**	**Obs**	**Exp**	**SMR**	**(95% CI)**	***N***	**Person-year**	**Obs**	**Exp**	**SMR**	**(95% CI)**
***(C81–C96) Lymphoid, hematopoietic and related tissues***												
Workers in the non-semiconductor division	4,512	56,730	1	2.0	0.49	(0.01 – 2.74)	4,392	55,544	0	0.8		
Office workers in the semiconductor division	13,079	172,998	5	5.9	0.85	(0.27 – 1.99)	3,092	37,250	0	0.6		
Operator	736	10,905	1	0.4	2.83	(0.07 – 15.79)	19,921	267,371	10	3.9	2.59	(1.24 – 4.76)*
Facility engineer	7,891	101,465	2	2.6	0.78	(0.04—2.80)	105	1,313	0	0.2		
Utility manager	911	13,878	0	0.4			25	235.9	0	0.004		
Process engineer	5,273	66,992	1	2.1	0.48	(0.01 – 2.68)	878	8,615	0	0.1		
Not classifiable	973	17,674	1	0.7	1.36	(0.03 – 7.58)	4,101	67,355	1	1.0	0.96	(0.02 – 5.34)
***(C91–C95) Leukemia***												
Workers in the non-semiconductor division	4,512	56,730	0	1.1			4,392	55,544	0	0.6		
Office workers in the semiconductor division	13,079	172,998	5	3.4	1.49	(0.48 – 3.48)	3,092	37,250	0	0.4		
Operator	736	10,905	1	0.2	4.82	(0.12 – 26.86)	19,921	267,371	8	2.7	2.92	(1.26 – 5.76)*
Facility engineer	7,891	101,465	2	1.7	1.15	(0.14 – 4.14)	105	1,313	0	0.01		
Utility manager	911	13,878	0	0.3			25	235.9	0	0.003		
Process engineer	5,273	66,992	1	1.2	0.80	(0.02 – 4.48)	878	8,615	0	0.1		
Not classifiable	973	17,674	1	0.4	2.60	(0.07 – 14.51)	4,101	67,355	0	0.7		
***(C82–C85) Non-Hodgkin’s lymphoma***												
Workers in the non-semiconductor division	4,512	56,730	1	0.7	1.46	(0.04 – 8.12)	4,392	55,544	0	0.1		
Office workers in the semiconductor division	13,079	172,998	0	2.0			3,092	37,250	0	0.1		
Operator	736	10,905	0	0.1			19,921	267,371	2	0.8	2.42	(0.29 – 8.75)
Facility engineer	7,891	101,465	0	0.7			105	1,313	0	0.004		
Utility manager	911	13,878	0	0.1			25	235.9	0	0.001		
Process engineer	5,273	66,992	0	0.7			878	8,615	0	0.03		
Not classifiable	973	17,674	0	0.3			4,101	67,355	1	0.2	4.03	(0.10 – 22.46)

*Obs* observed number of deaths, *Exp* expected number of deaths, *SMR* standardized mortality ratio, *CI* confidence interval

^*^p < 0.05

A sensitivity analysis was performed after excluding workers with less than 2 years of working duration (Additional files 2, 3 and 4). A total of 65,764 workers were retained, 18 of whom were excluded from the main analysis. Among female operators, the SMRs for malignant neoplasms in the lymphoid, hematopoietic, and related tissues and leukemia were 2.59 (95% CI = 1.24–4.76) and 2.92 (95% CI = 1.26–5.76), which were robust and supported the results of the main analysis.

Finally, we calculated the SMRs and 95% CIs for the malignant neoplasms in the lymphoid, hematopoietic, and related tissues among female operators based on the workers’ first working year (Additional file 5). Female operators who were employed in 2005 or later did not have significantly higher SMR for leukemia, but female operators were employed before 2005 had a significantly higher SMR for leukemia (14.5) (95% CI = 5.8–29.9).

**Fig. 2:**
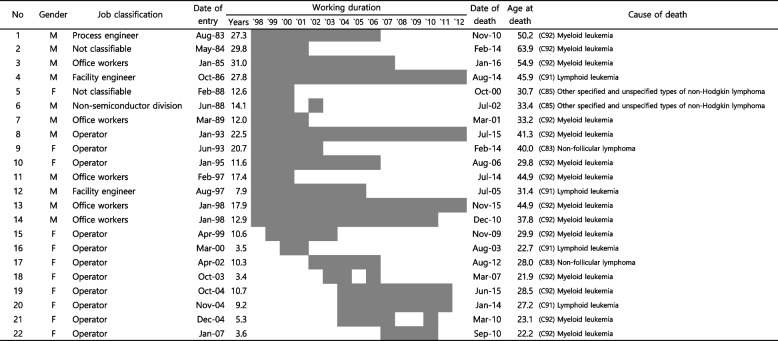
Characteristics of the observed deaths due to malignant neoplasms in the lymphoid, hematopoietic, and related tissues (C81–C96). The Job classification were as follows: workers in the non-semiconductor division (working in divisions other than the semiconductor division, such as the hard disk drive division, optical and mechanical solution division, and storage division), office workers in the semiconductor division, operator (operating facilities to produce semiconductor products or testing for acceptance and inspecting defective products), facility engineer (being assigned to specific manufacturing facilities for inspection and maintenance), utility manager (establishing and providing infrastructure for semiconductor production at the “whole-plant level”), process engineer (maintaining and improving the manufacturing process to improve the yield and quality of products), and not classifiable (cannot be included anywhere among the other six categories). If an employee’s job classification changed during the follow-up period, the longest-worked job classification was used as the representative category. The term “years” was defined as the years between the date of entry and the date of death

The causes of death among the ten female operators who had malignant neoplasms in the lymphoid, hematopoietic, and related tissues were as follows: myeloid leukemias (C92): six workers, lymphoid leukemias (C91): two workers, and non-follicular lymphomas (C83): two workers. The characteristics of the 22 participants who died due to malignant neoplasms in the lymphoid, hematopoietic, and related tissues are shown in Fig. 2. Among the 22 participants who died due to lymphohematopoietic cancer, 11 (50.0%) were male workers and 11 (50.0%) were female workers. Operator (*n* = 11, 50.0%) was the most common job classification of the 22 participants who died due to lymphohematopoietic cancer. The date of entry was between August 1983 and January 2007, while the average working duration was 11.4 years (SD ± 7.2). The mean age at death was 35.7 years (SD ± 11.4), and the most common cause of death was myeloid leukemia (*n* = 14, 63.6%).

## Discussion

Female operators working in the semiconductor industry in South Korea experienced excess cancer mortality due to malignant neoplasms in the lymphoid, hematopoietic, and related tissues, especially leukemia. Female operators showed a 2.59-fold increased risk of mortality due to malignant neoplasms in the lymphoid, hematopoietic, and related tissues compared with the general population and 2.92-fold increased risk of mortality due to leukemia.

To the best of our knowledge, this study is the first to report a significantly higher SMR for malignant neoplasms in the lymphoid, hematopoietic, and related tissues. Since the invention of the integrated circuit in 1959, Silicon Valley in California emerged as a high-tech industrial area through the 1960–1970s, when semiconductor enterprises such as Fairchild, Intel, and Advanced Micro Devices started their businesses. In the late 1970s, occupational health risks in the semiconductor industry began receiving attention. In 1983, Joseph Ladou raised concern over the high rate of illness and percentage of systemic poisoning among workers in the semiconductor manufacturing industry in California [22]. The Semiconductor Industry Study done in 1981 concluded that many toxic materials and a variety of solvents, acids, and metals, such as arsenic, are used in the semiconductor manufacturing process [23].

A key event that triggered health concerns in the semiconductor industry was a series of lawsuits filed by employees of the International Business Machine (IBM) in the 1990s. These employees claimed that their cancer was linked to chemical exposures in IBM's semiconductor and disk drive plants. Thus, epidemiological studies were conducted in the late 1990s and early 2000s examining the health risks associated with work in the semiconductor industry. Clapp et al. investigated cancer mortality among employees who worked in the IBM Endicott plant between 1969 and 2001. The study reported that the proportional cancer mortality ratio of all lymphatic and hematopoietic tissues was 1.23 times higher than that of the standard population in male workers [24]. Bender et al. investigated the employees of IBM’s semiconductor manufacturing facilities. They reported that the standardized incidence ratios (SIRs) for all cancer types (SIR = 0.81, 95% CI = 0.77–0.85), NHL (SIR = 0.94, 95% CI = 0.49–0.98), and leukemia (SIR = 0.70, 95% CI = 0.49–0.98) were not significantly higher. Other cohort studies reported significantly lower SMRs for all cancer types. Using the data of two large US semiconductor companies between 1968 and 2002, with 37,225 fabrication workers and 62,856 non-fabrication workers, compared to the general population, the SMR for all cancers was significantly low in fabrication workers (SMR = 0.74, 95% CI = 0.66–0.83) and non-fabrication workers (SMR = 0.72, 95% CI = 0.66–0.79). Similarly, the SMRs for all leukemia and aleukemia were also not significantly higher among fabrication workers (SMR = 0.75, 95% CI = 0.34–1.23) and non-fabrication workers (SMR = 0.82, 95% CI = 0.52–1.22) [15]. Despite the high level of public concern and the resentment of victims, it had been nearly impossible to suggest a causal relationship between the hundreds of cases of cancer and the work environment, especially for hematopoietic malignant disorders.

A 1985 study on cancer risk in semiconductor wafer processing workers was reported for the first time in the U.K. and has been reported across a series of cohort studies in the West Midlands. A significantly increased risk of some types of cancer was reported [25–27]. Using the standardized registration ratios (SRRs), the cancer incidence is calculated as the ratio of observed to expected number of cases based on the cancer incidence rates in England and Wales from 1971 to 2000. Results showed a significant excess morbidity for cancer of the rectum (SRR = 1.99, 95% CI = 1.20–3.10) and malignant melanoma (SRR = 2.17, 95% CI = 1.12–3.79). McElvenny et al. reported the cancer incidence and mortality rates among current and former workers at a Scottish semiconductor manufacturing facility, including 2,126 male workers and 2,262 female workers. In this cohort study, a significantly increased SMR for all types of malignant neoplasms or SRR for all cancer types were not found [12]. The SRR for malignant neoplasms of the respiratory and intrathoracic organs was significantly increased in all female workers (SRR = 2.73, 95% CI = 1.36–4.88) and female workers in fabrication areas (SRR = 3.17, 95% CI = 1.45–6.02). The mortality and incidence of hematopoietic cancers did not increase in male or female workers. Follow-up studies reported that there was no significant increase in the incidence and mortality rates of lung cancer [28, 29]. The studies reported in the UK could not identify the relationship between semiconductor processes and diseases. Furthermore, there was no significantly increased incidence or mortality for lymphohematopoietic cancers.

This occupational health issue emerged in Asian countries, including Taiwan, Singapore, and Korea, in the 2000s, when it had already been a social issue in the 1980s in the US and the 1990s in the UK [30]. The Occupational Safety and Health Research Institute of South Korea conducted a retrospective cohort study of 113,443 workers from 11 semiconductor companies between 1998 and 2008 [7]. The SMRs for all-cause mortality were 0.25 (95% CI = 0.21–0.29) in male workers and 0.66 (95% CI = 0.55–0.80) in female workers, and the SMR for malignant neoplasms in the lymphoid, hematopoietic, and related tissues in female workers was 1.56 (95% CI = 0.78–2.78). The SIRs for all cancer types were 0.86 (95% CI = 0.74–0.98) in male workers and 0.88 (95% CI = 0.74–1.03) in female workers. Among female workers, the SIR for malignant neoplasms in the lymphoid, hematopoietic, and related tissues was 1.54 (95% CI = 0.98–2.31), with a significantly higher SIR for NHL of 2.31 (95% CI = 1.23–3.95).

Our study reported a significant increase in lymphohematopoietic cancers among semiconductor workers, especially female operators. Several decades after this problem was identified across continents and countries, epidemiological evidence has been found supporting that the industry could increase the risk of lymphohematopoietic cancer. The semiconductor industry, historically, has faced challenges in occupational health due to the use of hazardous materials and chemicals [22]. Without definitive scientific proof of harm, the precautionary principle dictates that companies should take proactive measures to ensure worker safety. These measures may include implementing rigorous safety protocols, providing adequate protective gear, and carrying out routine health check-ups for workers [31, 32]. However, in the case of the semiconductor industry, protecting workers' health was not given top priority due to the rapidly changing industrial environment and the introduction of new processes. These instances underscore the importance of the precautionary principle in occupational health, emphasizing proactive action to prevent harm rather than reactive measures after damage occurrence.

Our study reported myeloid leukemia, lymphoid leukemia, and non-follicular lymphoma among female operators in the wafer fabrication process who experienced excess mortality from malignant lymphoid, hematopoietic, and related tissues. The female operators died in their 20 s. The age distribution of this group is notable; they were classified as “healthy workers,” and their SMRs for all-cause mortality were significantly lower than the general population in both sexes. Leukemia, especially acute myeloid leukemia, occurs in older adults, with a reported median age ranging from 63 to 71 years [33, 34]. The demographic characteristics of the participants in our study were unusual for individuals with this type of cancer, implying that there may be a cancer cluster in this industry [35]. Although it was difficult to determine whether a definite exposure to carcinogenic chemicals had occurred, recent studies reported that some substances can emit benzene during a chemical reaction, which could lead investigators to infer an association between the semiconductor industry in South Korea and leukemia [7, 8, 36].

The semiconductor industry uses numerous chemicals; however, information on the chemicals that are potentially harmful to workers is limited. In 1981, the Semiconductor Industry Study reported many toxic materials and gases such as arsine, phosphine, and diborane [23]. The semiconductor industry is high-tech, competitive, and fast-growing, so the work condition and environment have changed rapidly. Because of secrecy, rapid change, and short history, workers may not notice the substances they use, and even experts could not fully understand the risks [36]. One of the potential risky chemical exposures is benzene. According to the International Agency for Research on Cancer monograph for chemical agents and the associated occupations, benzene, 1,3-butadiene, formaldehyde, and rubber have been suggested as carcinogens, whose hazardous effects have been sufficiently demonstrated in human studies [37]. The chemicals used in industries, such as TCE, could contain benzene due to the limited purity of the substances [38, 39]. Considering that a low-level benzene exposure of < 10 ppm-year shows a relative risk of 2.2 (95% CI = 1.1–4.2) among benzene-exposed Chinese, the exposure to benzene during a past time could be a possible plausible mechanism for the occurrence of excess mortality due to malignant neoplasms in the lymphoid, hematopoietic, and related tissues. Although the accurate duration of exposure needs to be determined in order to estimate the period of cumulative exposure to benzene [40], a threshold effect between benzene and leukemia implies that high-intensity exposure could be related to a shorter exposure period [41]. Furthermore, as workers in the semiconductor industry could have been exposed to multiple chemical mixtures of more than 500 chemicals, there are limitations to the approach investigating the simple association between exposure to specific risk factors and a specific disease [42]. However, in this study, the insufficient information regarding the level of exposure to potential risk factors and the short exposure window were obstacles to determining and concluding the causal relationship between work environments and the observed excess mortality due to leukemia.

Our study has several strengths. First, information on the changes in the workplace and job classification was obtained from the company. Therefore, the job categories can be classified accurately and completely. Second, as the target population consisted of employees from one semiconductor company, we could minimize the difficulty in interpreting the results compared with the results of employees from different companies.

However, our study has several limitations. First, although we constructed a cohort of 65,782 workers with 878,325 person-years, which is the largest population of employees in the semiconductor industry in a single company, our study has limited statistical power for determining the incidence of rare malignancies such as those occurring in the lymphoid, hematopoietic, and related tissues. Calculating the SMR and SIR is not recommended when only five or fewer cases are observed as the results are considered unreliable [43]. Although we observed ten female operators who died from lymphohematopoietic cancer, special attention should be paid to the interpretation of SMRs for malignant neoplasms in the lymphoid, hematopoietic, and related tissues. Second, the causes of death were possibly misclassified. However, the classification of the causes of death according to the NSO data had an overall accuracy rate of 91.9%, and the causes of death were commonly misclassified as “unusual, unnatural death.” The reliability of the causes of cancer-related deaths was higher than that of other causes [44]. Third, information on the changes in the working environment is lacking. Although the SMRs for malignant neoplasms in the lymphoid, hematopoietic, and related tissues increased, more detailed assessments such as the job exposure matrices could not be carried out in our study. Fourth, we classified workers according to the job they had worked in for most years in cases of shifting job types. Therefore, information on the workers’ jobs during the study period was summarized. Although this could have attenuated our findings, we believe that the direction of the attenuation was null. Fifth, leukemia is a group of different disease subtypes, including acute myeloid leukemia, acute lymphoblastic leukemia, chronic lymphocytic leukemia, and chronic myeloid leukemia [45, 46]. However, the reference mortality by 5-year age and sex group was not provided at the subcategory level; hence, we could not calculate the SMR for myeloid leukemia or lymphoid leukemia separately. Finally, the electronic human resource records from the company only included employee’s information from 1998 and beyond. The company’s semiconductor factory has continuously expanded and been renovated since the establishment of the production line at Giheung, Korea in 1983. Considering that the work environment and protective measures implemented to avoid exposure from hazardous chemicals have continuously improved in the company, more workers died from malignant neoplasms in the lymphoid, hematopoietic, and related tissues. Workers who developed work-related diseases may have left before the year 1998, leading to a potential selection bias in terms of the healthy worker survivor effect. The lack of human resource records for subcontractor employees who are potentially exposed to hazardous work environments is another limitation of our study. Additionally, we hope that social discussions incorporate an intersectional perspective. The semiconductor industry predominantly comprises educated female workers in their twenties. It is possible that susceptibility to occupational diseases may be related to social factors. While this epidemiological study does not focus on this specific aspect, we believe that understanding the social context is crucial for societal improvement and the prevention of occupational diseases.

## Conclusion

A previous work environment for the semiconductor wafer fabrication process could be associated with an increased SMR for hematopoietic cancers and leukemia. However, the broad confidence interval of the SMRs, owing to the small number of observed and expected deaths, make it difficult to interpret the results. This study will be used as epidemiologic evidence of the workers’ compensation and serve as a basis for future epidemiologic studies to assess the potential health effects of working in a semiconductor wafer fabrication process industry among semiconductor workers.

## Supplementary Information

**Additional file 1: **The description of job categories of the participants who worked in a semiconductor company.

**Additional file 2: **Characteristics of South Korean workers in the a semiconductor factories among those who followed up more than 2 years during 1998–2014.

**Additional file 3: **Observed and expecteddeaths, SMRs, and 95% CIs for all-causes and malignant neoplasms among those who followed up more than 2 years during 1998–2014.

**Additional file 4: **Observed and Expected Deaths, SMRs, and 95% Cls for Malignant Neoplasms of Lymphoid, Hematopoietic and Related Tissues (C81-C96) by job classification.

**Additional file 5: **Observed and Expected Deaths, SMRs, and 95% Cls for Malignant Neoplasms of Lymphoid, Hematopoietic and Related Tissues (C81-C96) among female operators, according to the entry year.

## Data Availability

The datasets generated and/or analyzed during the current study are not publicly available but are available from the corresponding author upon reasonable request.

## Abbreviations

ICD-10: International statistical classification of diseases and related health problems, 10th revision
NHL: Non-Hodgkin’s lymphoma
NSO: National statistical office
SD: Standard deviation
SIR: Standardized incidence ratio
SMR: Standardized mortality ratios
SRR: Standardized registration ratio
UK: United Kingdom
US: United States
