# Supplementary material for: Lymphohematopoietic cancer mortality among Korean semiconductor manufacturing workers
Source: BMC Public Health. 2023 Aug 2;23:1473. doi: 10.1186/s12889-023-16325-z (PMC10398905; doi:10.1186/s12889-023-16325-z)
Supplement: Supplementary file 1 — Additional file 1: The description of job categories of the participants who worked in a semiconductor company. [file 12889_2023_16325_MOESM1_ESM.docx]

**Additional file 1. The description of job categories of the participants who worked in a semiconductor company.**

| **Classification** | **Description** |
| --- | --- |
| Workers in non-semiconductor division | working in divisions other than the semiconductor division, such as the hard disk drive division, optical and mechanical solution division, and storage division |
| Office workers in semiconductor division | Office workers not directly involving the manufacturing process |
| Operator | operating facilities to produce semiconductor products or testing for acceptance and inspecting defective products |
| Facility engineer | being assigned to specific manufacturing facilities for inspection and maintenance |
| Utility management | establishing and providing infrastructure for semiconductor production at the 'whole-plant level' |
| Process engineer | maintaining and improving the manufacturing process to improve the yield and quality of products |
| Not classifiable | cannot be included anywhere among the other six categories |
