# Supplementary material for: Lymphohematopoietic cancer mortality among Korean semiconductor manufacturing workers
Source: BMC Public Health. 2023 Aug 2;23:1473. doi: 10.1186/s12889-023-16325-z (PMC10398905; doi:10.1186/s12889-023-16325-z)
Supplement: Supplementary file 2 — Additional file 2: Characteristics of South Korean workers in the a semiconductor factories among those who followed up more than 2 years during 1998–2014. [file 12889_2023_16325_MOESM2_ESM.docx]

**Additional file 2. Characteristics of South Korean workers in the a semiconductor factories among those who followed up more than 2 years during 1998–2014.**

|  | Total | |  | Male | |  | | Female | |  |
| --- | --- | --- | --- | --- | --- | --- | --- | --- | --- | --- |
|  | n | (%) |  | n | (%) | |  | n | (%) | |
| Total | 65,764 | (100.0) |  | 33357 | (100.0) | |  | 32,407 | (100.0) | |
| Birth year |  |  |  |  |  | |  |  |  | |
| 1930–1939 | 1 | (0.001) |  | 1 | (0.003) | |  | 1 | (0.003) | |
| 1940–1949 | 37 | (0.1) |  | 37 | (0.1) | |  | 0 | (0) | |
| 1950–1959 | 687 | (1.0) |  | 685 | (2.1) | |  | 2 | (0.01) | |
| 1960–1969 | 5,813 | (8.8) |  | 5,724 | (17.2) | |  | 89 | (0.3) | |
| 1970–1979 | 22,361 | (34.0) |  | 14,936 | (44.8) | |  | 7,425 | (22.9) | |
| 1980–1989 | 34,095 | (51.8) |  | 11,892 | (35.7) | |  | 22,203 | (68.5) | |
| 1990–1999 | 2,770 | (4.2) |  | 82 | (0.3) | |  | 2,688 | (8.3) | |
| Job classification |  |  |  |  |  | |  |  |  | |
| Workers in non-semiconductor division | 8,901 | (13.5) |  | 4,510 | (13.5) | |  | 4,391 | (13.6) | |
| Office workers in semiconductor division | 16,167 | (24.6) |  | 13,075 | (39.2) | |  | 3,092 | (9.5) | |
| Operator | 20,655 | (31.4) |  | 735 | (2.2) | |  | 19,920 | (61.5) | |
| Facility engineer | 7,996 | (12.2) |  | 7,891 | (23.7) | |  | 105 | (0.3) | |
| Utility management | 936 | (1.4) |  | 911 | (2.7) | |  | 25 | (0.1) | |
| Process engineer | 6,149 | (9.4) |  | 5,271 | (15.8) | |  | 878 | (2.7) | |
| Not classifiable | 4,960 | (7.5) |  | 964 | (2.9) | |  | 3.996 | (12.3) | |
