# Supplementary material for: Lymphohematopoietic cancer mortality among Korean semiconductor manufacturing workers
Source: BMC Public Health. 2023 Aug 2;23:1473. doi: 10.1186/s12889-023-16325-z (PMC10398905; doi:10.1186/s12889-023-16325-z)
Supplement: Supplementary file 3 — Additional file 3: Observed and expecteddeaths, SMRs, and 95% CIs for all-causes and malignant neoplasms among those who followed up more than 2 years during 1998–2014. [file 12889_2023_16325_MOESM3_ESM.docx]

**Additional file 3. Observed and expected deaths, SMRs, and 95% CIs for all-causes and malignant neoplasms among those who followed up more than 2 years during 1998–2014.**

|  | Male | | | |  | Female | | | |
| --- | --- | --- | --- | --- | --- | --- | --- | --- | --- |
| Disease group (ICD-10) | Obs | Exp | SMR | 95% CI |  | Obs | Exp | SMR | 95% CI |
| All-cause | 158 | 697.9 | 0.23^*^ | (0.19 – 0.26) |  | 112 | 168.8 | 0.67^*^ | (0.55 – 0.80) |
| [C00-C97] Malignant neoplasm | 57 | 153.7 | 0.37^*^ | (0.28 – 0.48) |  | 47 | 39.3 | 1.20 | (0.88 – 1.59) |
| [C81-C96] Lymphoid, hematopoietic and related tissues | 11 | 14.1 | 0.78 | (0.39 – 1.40) |  | 11 | 6.4 | 1.71 | (0.85 – 3.06) |
| (C91-C95) Leukemia | 10 | 8.3 | 1.20 | (0.58 – 2.21) |  | 8 | 4.5 | 1.77 | (0.76 – 3.49) |
| (C82-C85) Non-Hodgkin’s lymphoma | 1 | 4.5 | 0.22 | (0.01 – 1.24) |  | 3 | 1.4 | 2.10 | (0.43 – 6.15) |

ICD-10, the International Classification of Diseases 10th revision; Obs, Observed number of deaths; Exp, Expected number of deaths; SMR, Standardized mortality ratio; CI, Confidence Intervals
