# Supplementary material for: Lymphohematopoietic cancer mortality among Korean semiconductor manufacturing workers
Source: BMC Public Health. 2023 Aug 2;23:1473. doi: 10.1186/s12889-023-16325-z (PMC10398905; doi:10.1186/s12889-023-16325-z)
Supplement: Supplementary file 4 — Additional file 4: Observed and Expected Deaths, SMRs, and 95% Cls for Malignant Neoplasms of Lymphoid, Hematopoietic and Related Tissues (C81-C96) by job classification. [file 12889_2023_16325_MOESM4_ESM.docx]

**Additional file 4. Observed and Expected Deaths, SMRs, and 95% Cls for Malignant Neoplasms of Lymphoid, Hematopoietic and Related Tissues (C81-C96) by job classification.**

|  | | Male | | | | | |  | | | | Female | | | | |  |
| --- | --- | --- | --- | --- | --- | --- | --- | --- | --- | --- | --- | --- | --- | --- | --- | --- | --- |
|  | **N** | | **Person-year** | **Obs** | **Exp** | **SMR** | **(95% CI)** | |  | **N** | **Person-year** | | **Obs** | **Exp** | **SMR** | **(95% CI)** | |
| ***[C81-C96] Lymphoid, hematopoietic and related tissues*** | | | | | | | | | | | | | | | | | |
| Workers in the non-semiconductor division | 4,510 | | 56,728 | 1 | 2.0 | 0.49 | (0.01 – 2.74) | |  | 4,391 | 55,542 | | 0 | 0.8 |  |  | |
| Office workers in the semiconductor division | 13,075 | | 172,993 | 5 | 5.9 | 0.85 | (0.28 – 1.99) | |  | 3,092 | 37,250 | | 0 | 0.6 |  |  | |
| Operator | 735 | | 10,903 | 1 | 0.4 | 2.83 | (0.07 – 15.79) | |  | 19,920 | 267,369 | | 10 | 3.9 | 2.59^*^ | (1.24 – 4.76) | |
| Facility engineer | 7,891 | | 101,465 | 2 | 2.6 | 0.78 | (0.09 - 2.80) | |  | 105 | 1,314 | | 0 | 0.2 |  |  | |
| Utility management | 911 | | 13,878 | 0 | 0.4 |  |  | |  | 25 | 236 | | 0 | 0.004 |  |  | |
| Process engineer | 5,271 | | 66,989 | 1 | 2.1 | 0.48 | (0.01 – 2.68) | |  | 878 | 8,615 | | 0 | 0.1 |  |  | |
| Not classifiable | 964 | | 17,671 | 1 | 0.7 | 1.36 | (0.03 – 7.58) | |  | 3.996 | 67,349 | | 1 | 1.0 | 0.96 | (0.02 – 5.35) | |
| ***(C91-C95) Leukemia*** |  | |  |  |  |  |  | |  |  |  | |  |  |  |  | |
| Workers in the non-semiconductor division | 4,512 | | 56,728 | 0 | 1.1 |  |  | |  | 4,392 | 55,542 | | 0 | 0.6 |  |  | |
| Office workers in the semiconductor division | 13,079 | | 172,993 | 5 | 3.4 | 1.49 | (0.48 – 3.48) | |  | 3,092 | 37,250 | | 0 | 0.4 |  |  | |
| Operator | 736 | | 10,903 | 1 | 0.2 | 4.82 | (0.12 – 26.86) | |  | 19,921 | 267,369 | | 8 | 2.7 | 2.92^*^ | (1.26 – 5.76) | |
| Facility engineer | 7,891 | | 101,465 | 2 | 1.7 | 1.15 | (0.14 – 4.14) | |  | 105 | 1,314 | | 0 | 0.01 |  |  | |
| Utility management | 911 | | 13,878 | 0 | 0.3 |  |  | |  | 25 | 236 | | 0 | 0.003 |  |  | |
| Process engineer | 5,273 | | 66,989 | 1 | 1.2 | 0.80 | (0.02 – 4.48) | |  | 878 | 8,615 | | 0 | 0.1 |  |  | |
| Not classifiable | 973 | | 17,671 | 1 | 0.4 | 2.60 | (0.07 – 14.51) | |  | 4,101 | 67,349 | | 0 | 0.7 |  |  | |
| ***(C82-C85) Non-Hodgkin’s lymphoma*** | | | | | | | | | | | | | | | | | |
| Workers in the non-semiconductor division | 4,512 | | 56,728 | 1 | 0.7 | 1.46 | (0.04 – 8.12) | |  | 4,392 | 55,542 | | 0 | 0.2 |  |  | |
| Office workers in the semiconductor division | 13,079 | | 172,993 | 0 | 2.0 |  |  | |  | 3,092 | 37,250 | | 0 | 0.1 |  |  | |
| Operator | 736 | | 10,903 | 0 | 0.1 |  |  | |  | 19,921 | 267,369 | | 2 | 0.8 | 2.42 | (0.29 – 8.75) | |
| Facility engineer | 7,891 | | 101,465 | 0 | 0.7 |  |  | |  | 105 | 1,314 | | 0 | 0.004 |  |  | |
| Utility management | 911 | | 13,878 | 0 | 0.1 |  |  | |  | 25 | 236 | | 0 | 0.001 |  |  | |
| Process engineer | 5,273 | | 66,989 | 0 | 0.7 |  |  | |  | 878 | 8,615 | | 0 | 0.03 |  |  | |
| Not classifiable | 973 | | 17,671 | 0 | 0.3 |  |  | |  | 4,101 | 67,349 | | 1 | 0.2 | 4.03 | (0.10 – 22.46) | |

Obs, Observed number of deaths; Exp, Expected number of deaths; SMR, Standardized mortality ratio; CI, Confidence Intervals

* *p* < 0.05
