# Supplementary material for: Lymphohematopoietic cancer mortality among Korean semiconductor manufacturing workers
Source: BMC Public Health. 2023 Aug 2;23:1473. doi: 10.1186/s12889-023-16325-z (PMC10398905; doi:10.1186/s12889-023-16325-z)
Supplement: Supplementary file 5 — Additional file 5: Observed and Expected Deaths, SMRs, and 95% Cls for Malignant Neoplasms of Lymphoid, Hematopoietic and Related Tissues (C81-C96) among female operators, according to the entry year. [file 12889_2023_16325_MOESM5_ESM.docx]

**Additional file 5. Observed and Expected Deaths, SMRs, and 95% Cls for Malignant Neoplasms of Lymphoid, Hematopoietic and Related Tissues (C81-C96) among female operators, according to the entry year.**

|  | | Male | | | | | |  | | | | Female | | | | |
| --- | --- | --- | --- | --- | --- | --- | --- | --- | --- | --- | --- | --- | --- | --- | --- | --- |
|  | **N** | | **Person-year** | **Obs** | **Exp** | **SMR** | **(95% CI)** | |  | **N** | **Person-year** | | **Obs** | **Exp** | **SMR** | **(95% CI)** |
| ***[C81-C96] Lymphoid, hematopoietic and related tissues*** | | | | | | | | | | | | | | | | |
| Before 2005 | 171 | | 1,492 | 1 | 0.04 | 28.1 | (0.7 – 156.8) | |  | 100 | 758 | | 9 | 0.7 | 13.1 | (6.0 – 24.9) |
| 2005 or after | 100 | | 759 | 0 | 0.02 |  |  | |  | 4,124 | 32,272 | | 1 | 0.4 | 2.3 | (0.1 – 12.8) |
| ***(C91-C95) Leukemia*** |  | |  |  |  |  |  | |  |  |  | |  |  |  |  |
| Before 2005 | 171 | | 1,492 | 1 | 0.03 | 38.9 | (0.9 – 216.8) | |  | 100 | 758 | | 7 | 0.5 | 14.5 | (5.8 – 29.9) |
| 2005 or after | 100 | | 759 | 0 | 0.01 |  |  | |  | 4,124 | 32,272 | | 1 | 0.3 | 3.3 | (0.1 – 18.2) |
| ***(C82-C85) Non-Hodgkin’s lymphoma*** | | | | | | | | | | | | | | | | |
| Before 2005 | 171 | | 1,492 | 0 | 0.01 |  |  | |  | 100 | 758 | | 2 | 0.1 | 14.8 | (1.8 – 53.5) |
| 2005 or after | 100 | | 759 | 0 | 0.005 |  |  | |  | 4,124 | 32,272 | | 0 | 0.1 |  |  |

Obs, Observed number of deaths; Exp, Expected number of deaths; SMR, Standardized mortality ratio; CI, Confidence Intervals

* *p* < 0.05
